# Supplementary figures and images for: Oncogenic KRAS alters splicing factor phosphorylation and alternative splicing in lung cancer
Source: BMC Cancer. 2022 Dec 16;22:1315. doi: 10.1186/s12885-022-10311-1 (PMC9756471; doi:10.1186/s12885-022-10311-1)

**A**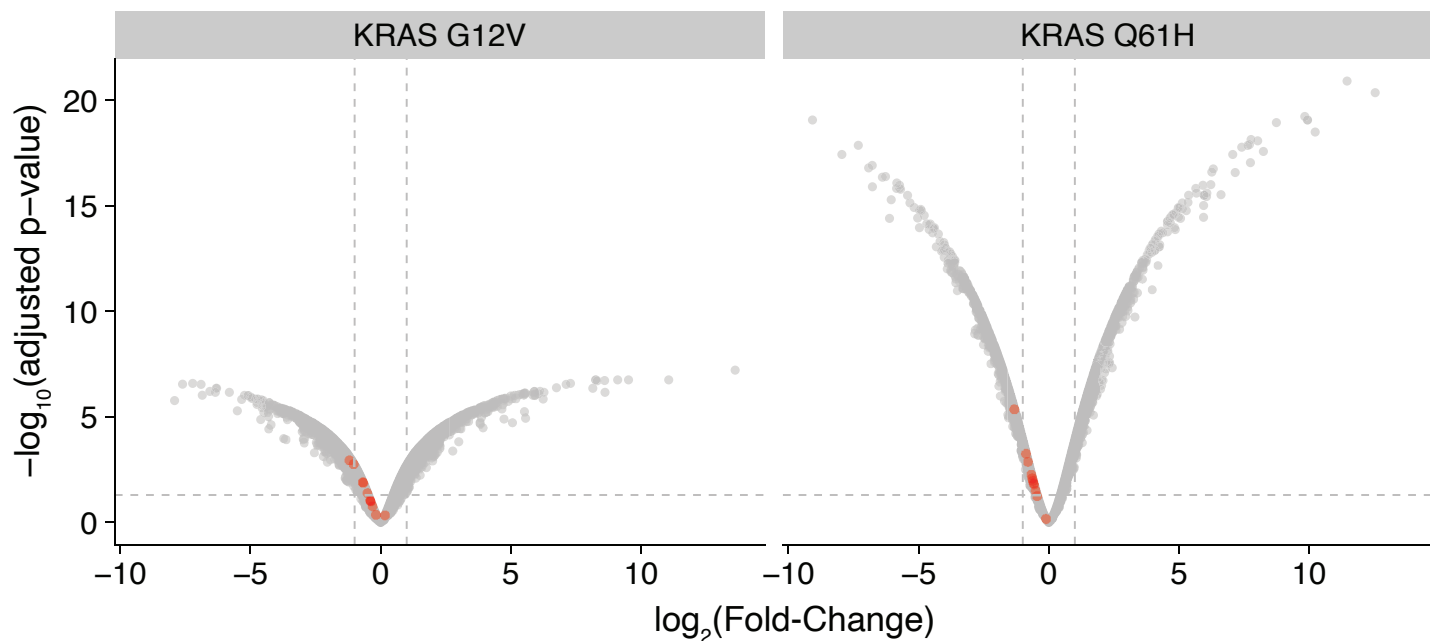**B**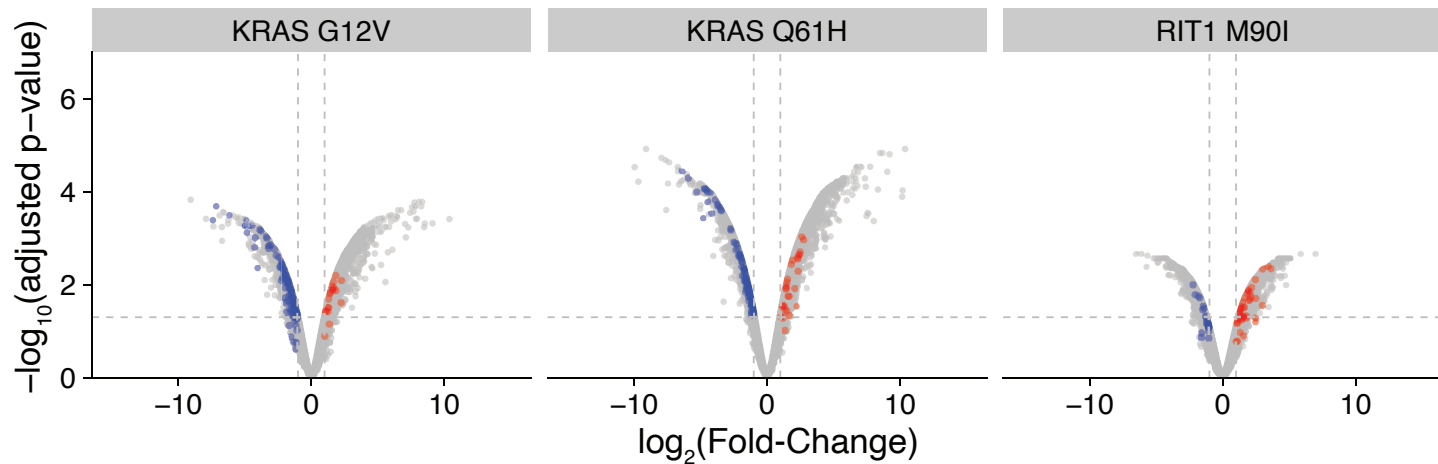

Supplement: Supplementary file 1 — Additional file 1: Supplementary Fig. 1. A) Volcano plot of differential protein abundance in KRASG12V and KRASQ61H cells compared to KRASWT cells. Labeled in red are SR proteins. B) Volcano plot of phosphorylation of phosphosites in KRASG12V and KRASQ61H cells compared to KRASWT cells, and RIT1M90I compared to RIT1WT. Labeled are phosphosites on proteins in the GO RNA SPLICING gene set which are downregulated (blue) or upregulated (red). [file 12885_2022_10311_MOESM1_ESM.pdf]

# Supplemental Figure 2

A

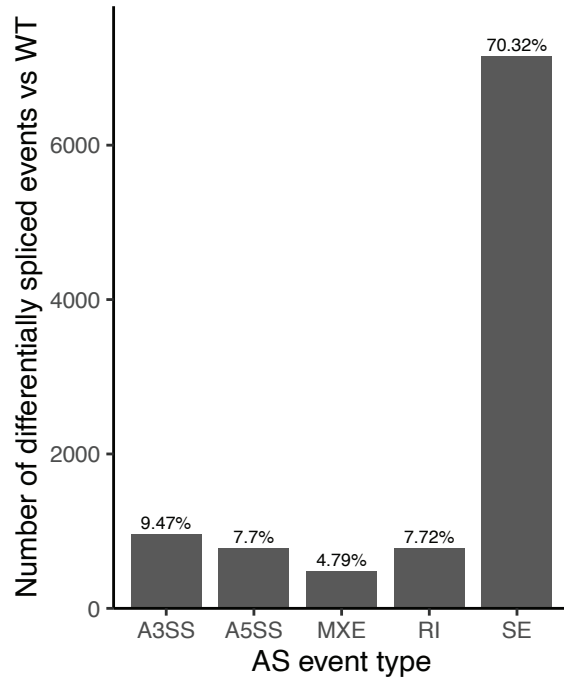

B

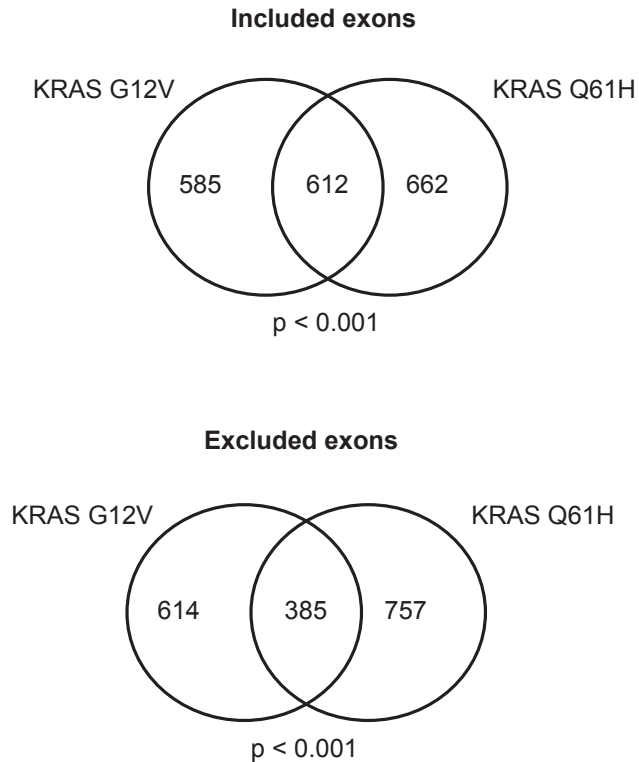

Supplement: Supplementary file 2 — Additional file 2: Supplementary Fig. 2. A) Number of differentially spliced events vs WT grouped by alternative splicing event type (A3SS = Alternative 3′ splice site; A5SS = Alternative 5′ splice site; MXE = Mutually exclusive exon; RI = Retained intron; SE = Skipped exon). Labeled percentages describe proportion of the group out of all differentially spliced events. B) Overlap of differentially spliced exons either more included (top) or more excluded (bottom) in KRASG12V vs KRASWT and KRASQ61H vs KRASWT, P-values calculated by modeling events as a hypergeometric distributions. [file 12885_2022_10311_MOESM2_ESM.pdf]

A

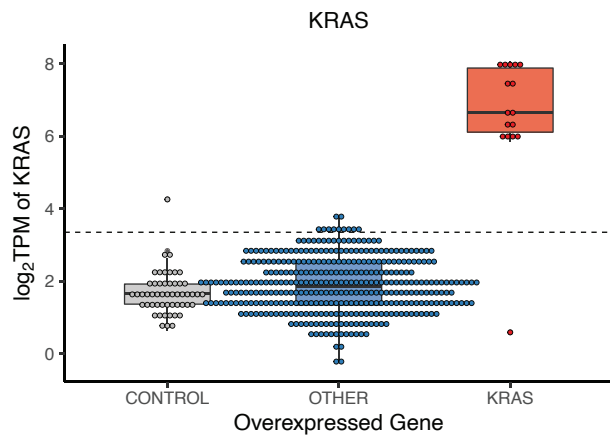

B

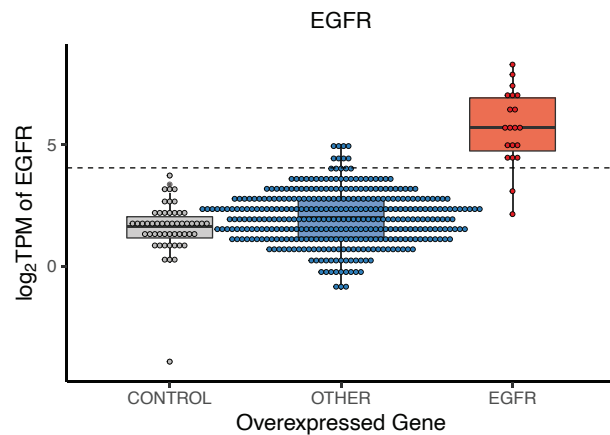

C

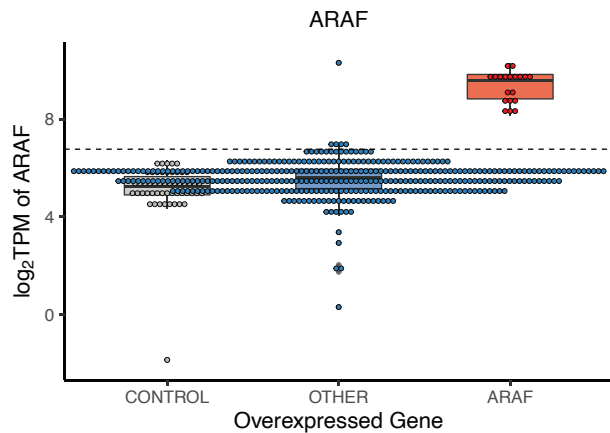

D

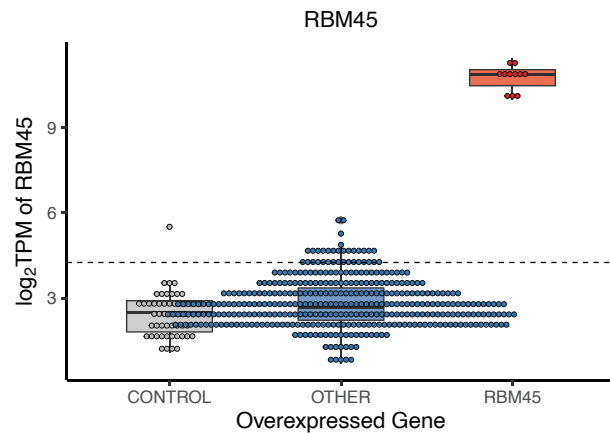

E

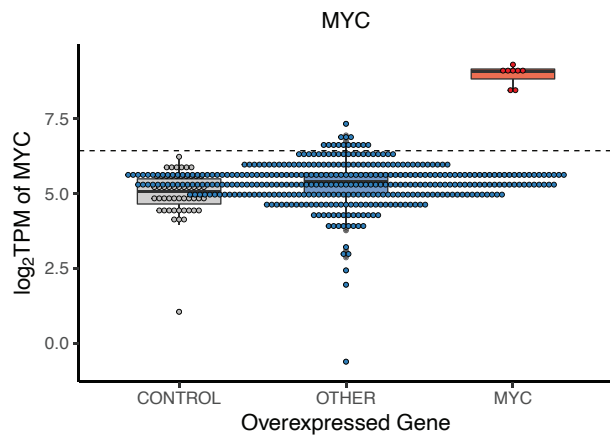

F

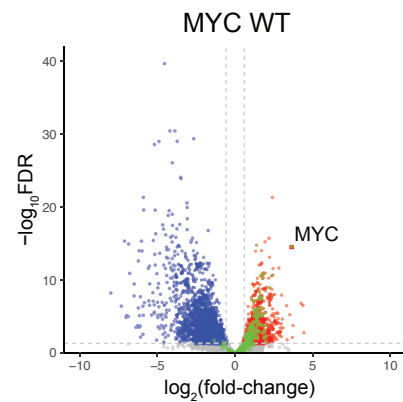

Supplement: Supplementary file 4 — Additional file 4: Supplementary Fig. 3. A) Volcano plot of differentially expressed genes in wild-type MYC overexpressing cells. Blue = downregulated transcripts. Red = upregulated transcripts. Green = transcripts in mSigDB hallmark gene sets MYC targets V1 and V2. B) mRNA expression levels of MYC in vector control cells (grey), cells overexpressing non-MYC alleles (blue), and cells overexpressing MYC alleles (red). C) Same as B) for KRAS. D) Same as B) for EGFR. E) Same as B) for ARAF. F) Same as B) for RBM45. [file 12885_2022_10311_MOESM4_ESM.pdf]

A

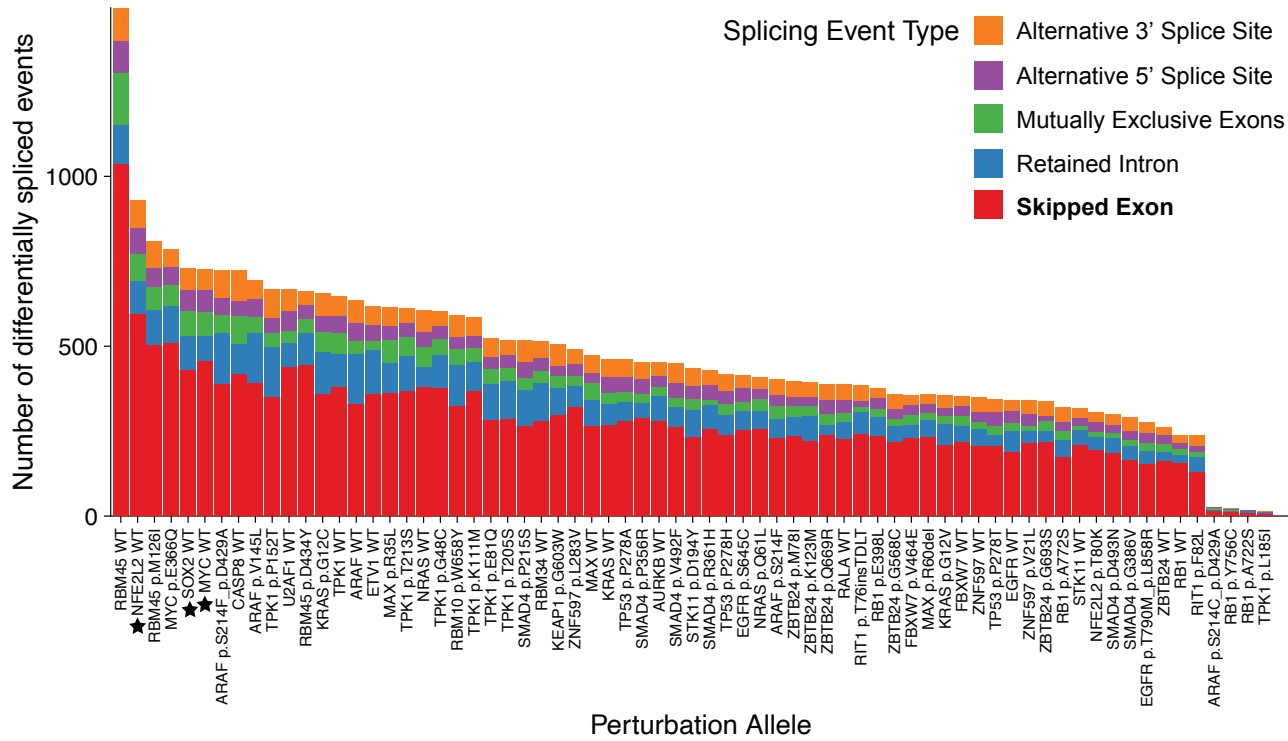

B

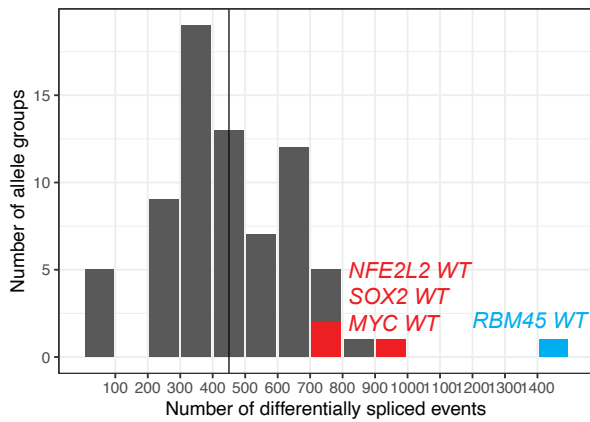

C

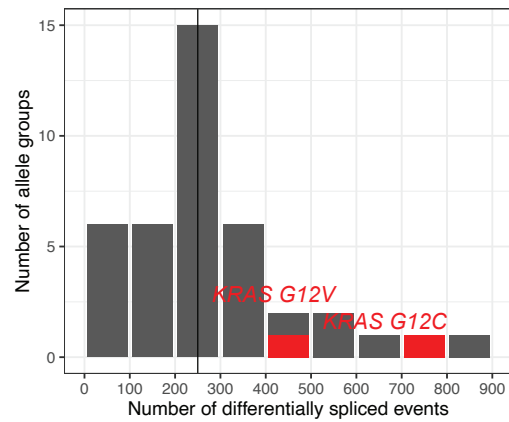

D

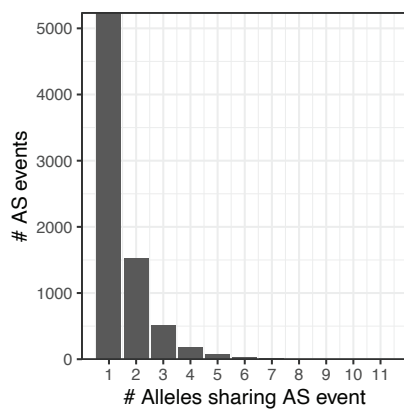

E

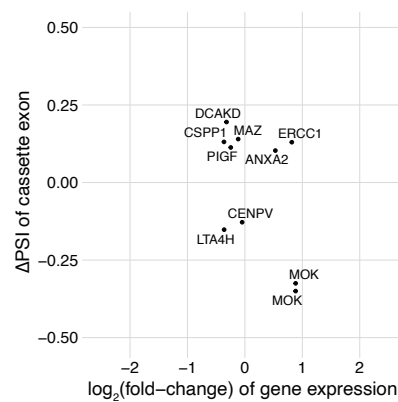

Supplement: Supplementary file 5 — Additional file 5: Supplementary Fig. 4. A) Number of events differentially spliced between cells with genetic perturbations and cells with vector controls. Colors represent 5 major alternative splicing event categories. Wild-type alleles of transcription factors are starred. B) Number of events differentially spliced between cells with genetic perturbations and cells with vector controls. Vertical line = mean number of events across screen. C) Number of events differentially spliced between cells with variant alleles and cells with respective wild-type alleles. Vertical line = mean number of events across screen. D) Number of differential alternative splicing (AS) events unique to one variant allele or overlapping between multiple alleles. E) Comparing change in Percent Spliced In (∆PSI) of cassette exons and the differential expression of the corresponding gene transcript. [file 12885_2022_10311_MOESM5_ESM.pdf]

**A**

**SF3B2**

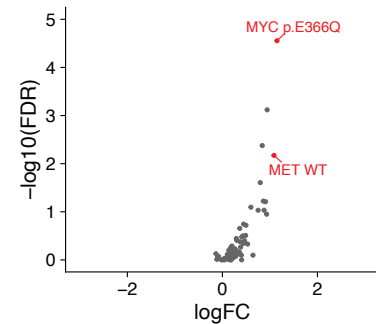

**SRSF1**

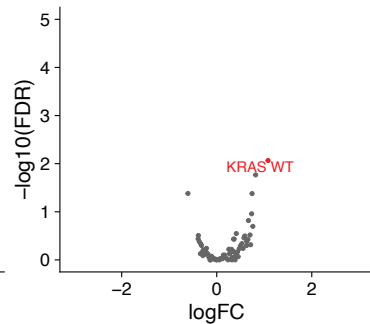

**SRSF2**

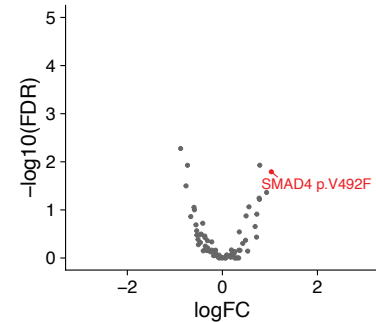

**SRSF7**

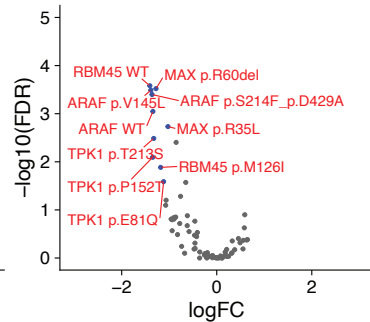

**B**

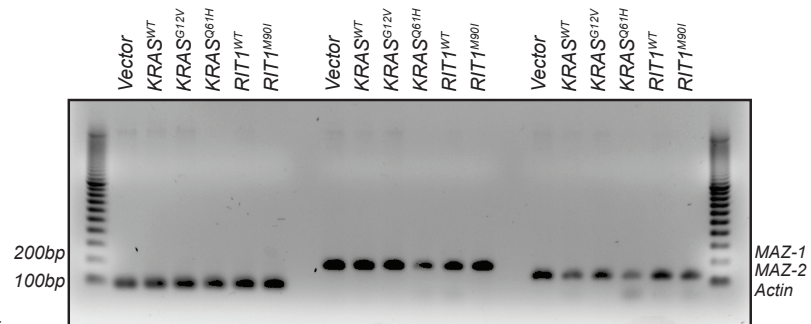

**C**

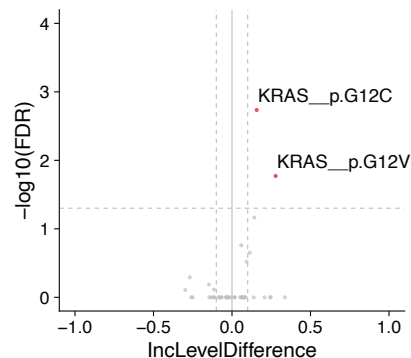

Supplement: Supplementary file 7 — Additional file 7: Supplementary Fig. 5. A) mRNA expression of splicing factors SF3B1, SRSF1, and SRSF7. B) End-point PCR gel detecting MAZ-1 and MAZ-2 isoforms in AALE cells overexpressing vector, KRAS, or RIT1 variants. C) Differential splicing of MAZ exon V′ in variant alleles compared to respective wild-type alleles. [file 12885_2022_10311_MOESM7_ESM.pdf]
